# Sorghum Phenolic Compounds Are Associated with Cell Growth Inhibition through Cell Cycle Arrest and Apoptosis in Human Hepatocarcinoma and Colorectal Adenocarcinoma Cells

**DOI:** 10.3390/foods10050993

**Published:** 2021-05-01

**Authors:** Xi Chen, Jiamin Shen, Jingwen Xu, Thomas Herald, Dmitriy Smolensky, Ramasamy Perumal, Weiqun Wang

**Affiliations:** 1 Department of Food Nutrition Dietetics and Health, Kansas State University, Manhattan, KS 66506, USA; xchen@whpu.edu.cn (X.C.); jiamins@ksu.edu (J.S.); jw-xu@shou.edu.cn (J.X.); 2 School of Food Science and Engineering, Wuhan Polytechnic University, Wuhan 430023, China; 3 College of Food Science and Technology, Shanghai Ocean University, Shanghai 201306, China; 4 USDA-ARS Grain Quality and Structure Research, 1515 College Ave, Manhattan, KS 66502, USA; Tom.Herald@ars.usda.gov (T.H.); dmiriy.smolensky@usda.gov (D.S.); 5 Agricultural Research Center, Kansas State University, Hays, KS 67601, USA; perumal@ksu.edu

**Keywords:** sorghum, phenolic compounds, cell growth inhibition, cell cycle analysis, apoptosis, HepG2, Caco-2

## Abstract

Phenolic compounds in some specialty sorghums have been associated with cancer prevention. However, direct evidence and the underlying mechanisms for this are mostly unknown. In this study, phenolics were extracted from 13 selected sorghum accessions with black pericarp while F10000 hybrid with white pericarp was used as a control, and cell growth inhibition was studied in hepatocarcinoma HepG2 and colorectal adenocarcinoma Caco-2 cells. Total phenolic contents of the 13 high phenolic grains, as determined by Folin–Ciocalteu, were 30–64 mg GAE/g DW in the phenolic extracts of various accessions compared with the control F10000 at 2 mg GAE/g DW. Treatment of HepG2 with the extracted phenolics at 0–200 μM GAE up to 72 h resulted in a dose- and time-dependent reduction in cell numbers. The values of IC_50_ varied from 85 to 221 mg DW/mL while the control of F10000 was 1275 mg DW/mL. The underlying mechanisms were further examined using the highest phenolic content of PI329694 and the lowest IC_50_ of PI570481, resulting in a non-cytotoxic decrease in cell number that was significantly correlated with increased cell cycle arrest at G2/M and apoptotic cells in both HepG2 and Caco-2 cells. Taken together, these results indicated, for the first time, that inhibition of either HepG2 or Caco-2 cell growth by phenolic extracts from 13 selected sorghum accessions was due to cytostatic and apoptotic but not cytotoxic mechanisms, suggesting some specialty sorghums are a valuable, functional food, providing sustainable phenolics for potential cancer prevention.

## 1. Introduction

Comparable with other cereals, the nutritional value of sorghum has been thought to be less than it actually is for many decades. Reference [1] suggests that people still underestimate the nutritional advantages of sorghum. It is well-known that sorghum is a gluten-free food and suitable for people with celiac disease. Some specialty sorghums have recently gained particular interest because of their high level of phenolics and potential health benefits, especially in cancer prevention [2,3,4,5]. Previous investigation has shown that an average of 35% of overall human cancer mortality is related to diet [6]. Sorghum can be a part of a plant-based healthy diet as certain accessions of sorghum contain many more bioactive phenolics than other crops, such as wheat, barley, rice, maize, rye, and oats [7]. Numerous studies have reported the cancer-preventive effects of phenolic compounds in fruits and vegetables, although few focused on specialty sorghum whole grain [1,8].

Compelling data from epidemiological and animal studies have suggested that phenolic compounds could potentially contribute to anti-cancer effects through their biological properties including antioxidant activity, induction of cell cycle arrest and apoptosis, and promotion of tumor suppressor proteins, etc. [9,10,11,12,13]. There is plenty of literature on the properties of various phenolic-rich foods such as tea and red wine/grapes in relation to various types of cancer [8], while studies regarding the association between sorghum phenolic compounds and cancers are scarce. Epidemiological studies have reported that sorghum consumption consistently correlates with a low incidence of esophageal cancer in various parts of the world (including several parts of Africa, Russia, India, China, Iran, etc.), whereas wheat and corn consumption correlates with an elevated incidence of esophageal cancer [10,11,12,13,14]. In vivo studies regarding the anti-cancer effects of sorghum phenolics are reported even less. Lewis et al. [15] reported in 2008 that feeding normolipidemic rats a diet containing sorghum bran could significantly reduce the number of aberrant crypts in the rats. More recently, Park et al. [16] found that the metastasis of breast cancer to the lungs was blocked by sorghum extracts in the immune-deficient mouse metastasis model. However, the mechanisms by which sorghum reduced the risk of cancer are unclear. A few in vitro studies published recently, using sorghum extracts to treat several cancer cells, including leukemia (HL-60) [17], breast (MCF-7, MDA-MB 231) [18,19], colon (HT-29) [19], and liver (HepG2) [19] cells, found consistent results in the induction of cell apoptosis, inhibition of cell proliferation, and promotion of the expression of cell cycle regulators.

Given the high potential of the benefits of sorghum phenolics and the lag in research when compared to other plant foods, black sorghum with high phenolic compounds is a candidate that deserves systematic investigation. Therefore, the present study investigated the effects of phenolics extracted from 13 specialty sorghum accessions on cancer cell growth in both hepatocarcinoma HepG2 and colorectal adenocarcinoma Caco2 cell lines. The underlying mechanisms regarding cytotoxicity, cell cycle interruption, and apoptosis induction were further examined.

## 2. Materials and Methods

### 2.1. Sorghum Accessions

Thirteen specialty sorghum accessions with black pericarp were selected based upon their high levels of phenolic content, including PI152653, PI152687, PI193073, PI329694, PI559733, PI559855, PI568282, PI570366, PI570481, PI570484, PI570819, PI570889 and PI570993. Another sorghum accession, F10000 with white pericarp, was selected as a control as it contains low levels of phenolics. All the sorghum accessions were provided by the Kansas State University-Agricultural Research Center, Hays, KS, USA.

### 2.2. Reagents

Reagents including acetone, ethanol, Folin–Ciocalteu reagent, gallic acid, sodium carbonate, HyClone Dulbecco’s Modified Eagle Medium (DMEM), fetal bovine serum, phosphate buffered saline (PBS), penicillin/streptomycin, trypsin-EDTA, propidium iodide (PI), and RNase were purchased from Fisher Scientific Co. L.L.C (Pittsburgh, PA, USA). CytoSelect^TM^ LDH Cytotoxicity Assay Kit was purchased from the Cell Biolabs, Inc. (San Diego, CA, USA).

### 2.3. Phenolic Extraction

Sorghum flour from each accession at 0.2–0.5 g was extracted in 10 mL of 70% aqueous acetone (*v*/*v*) for 2 h while shaking at low speed using a 211DS shaking incubator (Labnet International Inc., Edison, NJ 08817, USA) at room temperature, followed by storage at −20 °C in the dark overnight allowing the phenolics to be completely diffused from the cellular matrix into the solvent. The extract was then equilibrated at room temperature and centrifuged at 2970× *g* for 10 min. The residue was rinsed with an additional 10 mL of solvents with 5 min of shaking and centrifuged at 2970× *g* for another 10 min. Both supernatants were combined, and two aliquots were used for either total phenolic content determination or cell culture treatment. The aliquot for cell culture treatment was dried under a stream of nitrogen, and then dissolved in dimethyl sulfoxide (DMSO) to make a stock solution at −20 °C. Before its use for cell culture treatment, the stock solution was diluted with a fresh medium to achieve the desired concentration at 0–200 μM GAE. The final DMSO concentration in each treatment was kept at 0.1% *v*/*v*, which did not alter cell growth or cell cycle measurements significantly when compared with the DMSO-free medium. All extractions and treatments were conducted in triplicate.

### 2.4. Total Phenolic Content

Total phenolic content was determined using a Folin–Ciocalteu assay [20]. A stock solution of gallic acid at 1 mg/mL in distilled water was prepared and the final concentrations ranging from 12.5 to 200 μg/mL in 70% acetone were diluted for a standard curve. To each of the 96 wells, 75 μL distilled water was added, followed by 25 μL of either an aliquot of extracts or gallic acid as a standard at various concentrations. Folin–Ciocalteu reagent diluted 1:1 with distilled water was added to each well. The reaction was allowed to stand for 10 min at room temperature, and then 100 μL of Na_2_CO_3_ solution at 7.5% (*w*/*v*) was added to each well. The plate was covered and left to stand in the dark for 90 min before measuring. Absorbance was read using a microplate reader Synergy HT, BioTek with Gen5^TM^ 2.0 data analysis software (Winnoski, VT, USA). Results were expressed as mg gallic acid equivalent (GAE) per g dry weight (DW).

### 2.5. Cell Culture

The human hepatocarcinoma HepG2 (HB-8065) and human colorectal adenocarcinoma Caco-2 (HTB-37) were purchased from the American Type Culture Collection, Manassas, VA 20108, USA. Cells were cultured in DMEM supplemented by 10% FBS, 100 μg/mL streptomycin, and 100 units/mL penicillin at 37 °C in a 5% CO_2_ humidified atmosphere. Cells in the exponential growth phase were used for all the experiments.

### 2.6. Cell Growth Inhibition Assay

Two milliliter cell suspensions (1 × 10^5^ cells/mL) were seeded into 6-well plates and cultured in a humidified incubator to allow adhesion. Cells were then treated with extracted sorghum phenolics at 0–200 μM GAE for up to 72 h. After incubation with each treatment, cells at 24, 48 and 72 h were, respectively, detached by 0.05% trypsin-EDTA solution at 37 °C and then suspended in PBS. The number of suspended cells was counted with a hemocytometer as described in our previous publication [21,22].

### 2.7. Cytotoxicity Assay

Cytotoxicity was assessed by lactate dehydrogenase (LDH) leakage into the culture medium. The activity of LDH in the medium was determined using a commercially available kit CytoSelect^TM^ LDH Cytotoxicity Assay Kit from Cell Biolabs, Inc. (San Diego, CA 92126, USA). Cell suspension containing 0.1–1.0 × 10^6^ cells/mL was seeded into a 96-well plate and cultured at 37 °C and 5% CO_2_ with sorghum extracts at 0–200 μM GAE. Negative control was applied using sterile water and positive control was applied by Triton X-100. Aliquots of media and reagents were mixed in a 96-well plate and incubated at 37 °C for 0.5 h. Absorbance was recorded using a microplate reader SynergyHT, BioTek (Winnoski, VT, USA), and analyzed with Gen5^TM^ 2.0 data analysis software. The % of relative cytotoxicity was calculated using the following equation

$$\frac{OD\left(experiment\;sample\right)-OD\left(negative\;control\right)}{OD\left(positive\;control\right)-OD\left(negative\;control\right)}\;\text{×}\;100=\%\;\mathrm{Relative}\;\mathrm{Cytotoxicity}$$

### 2.8. Cell Cycle Analysis

Cell cycle analysis was conducted according to our previous publication [21]. After treatment, cells were detached and fixed in 70% ethanol at 4 °C. Cells were then re-suspended in 20 μg/mL of propidium iodide (PI) staining solution with 5 U/mL RNase at 37 °C for 15 min before analysis by a flow cytometry (LSRFortessa X-20 and FACSCalibur, BD, Franklin Lakes, NJ, USA) with excitation at 488 nm and emission at 617 nm.

### 2.9. Apoptosis Analysis

Apoptosis analysis was conducted according to our previous publication [22]. Briefly, treated cells were collected and fixed by 1% paraformaldehyde and 70% ice cold ethanol at a concentration of 1–2 × 10^6^ cells/mL. The cells were stored at −20 °C for several days. Fixed cells were analyzed for apoptosis by FITC annexin V staining protocol according to commercial instructions (BioLegend Inc., San Diego, CA, USA).

### 2.10. Statistics

Data were analyzed by the SAS statistical system (version 9.2). The significance of the trend for cell growth inhibition at various concentrations for three exposure times was analyzed with linear regression. The effect of extracted phenolic at various concentrations for 72 h on cell cycle and apoptosis was analyzed by one-way ANOVA with Tukey adjustment. Pearson correlation coefficients (r) were used to analyze the relationships between total phenolic contents and IC_50_ values for all sorghum accessions, as well as cell numbers and percentages of cell arrest at G2/M or apoptosis.

## 3. Results

### 3.1. Total Phenolic Content

As shown in Table 1, total phenolic contents of the 13 specialty sorghum accessions ranged from 31 to 63.7 mg GAE/g DW, with the highest being PI329694 and the lowest being the control F10000.

### 3.2. Cell Growth Inhibition

**Figure 1:**
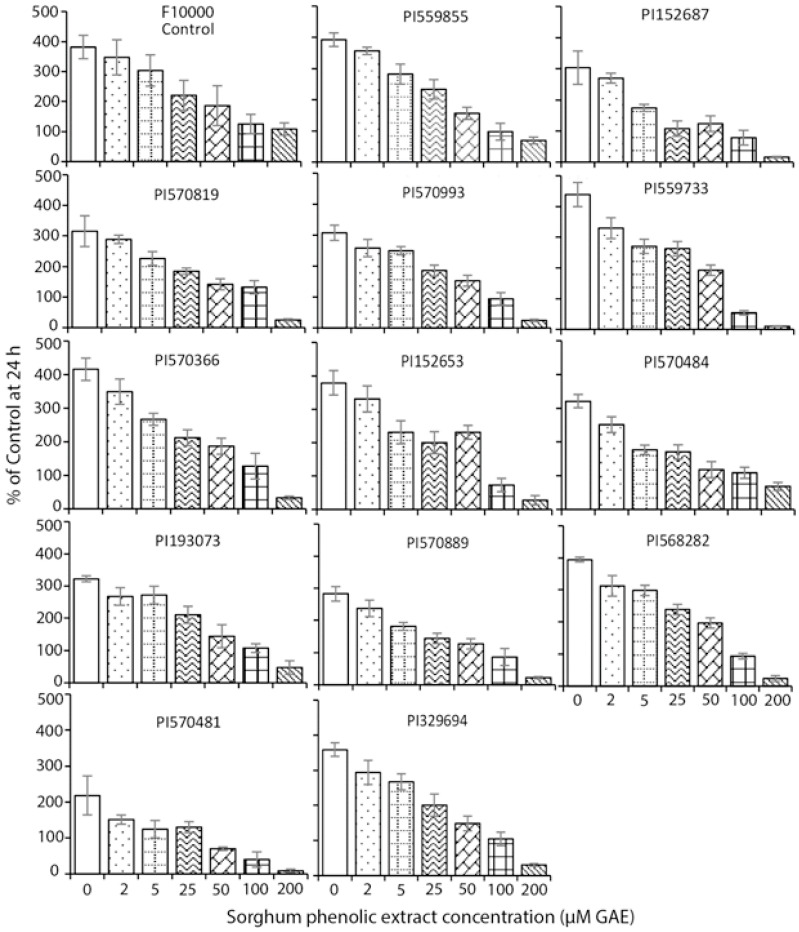
The effect of various sorghum phenolic extracts at 0–200 μM GAE for up to 72 h on cell growth in HepG2 cells. HepG2 cells were cultured with phenolic extracts at various concentrations (0–200 μM GAE) for up to 72 h in 6-well plates, then detached by trypsin-EDTA solution, and the cell number was counted by a hemacytometer. Values are expressed as Mean ± SD (*n* = 3). The significance of the trend for cell growth inhibition at various concentrations was analyzed with linear regression, *p* < 0.05.

As shown in Figure 1, treatment of HepG2 with the extracted phenolics at 0–200 μM GAE up to 72 h resulted in a dose- and time-dependent reduction in cell number. The values of IC_50_ varied from 85 to 221 mg DW/mL, while the control of F10000 was 1275 mg DW/mL (Table 2). The lowest IC_50_ value was observed in the phenolic extract from PI570481, suggesting PI570481 to be the most potent in suppressing HepG2 cell growth.

**Figure 2:**
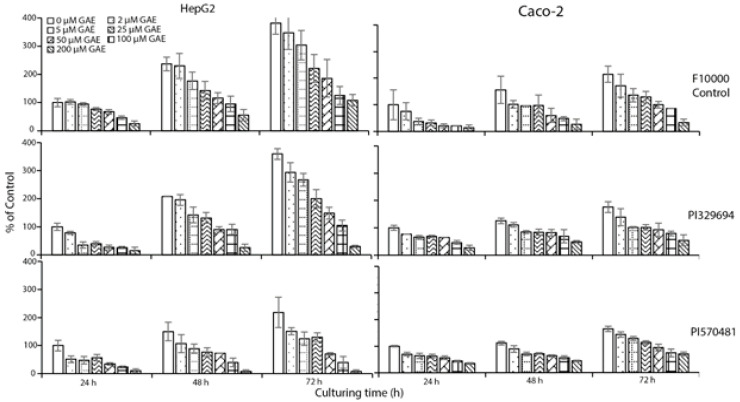
The effect of representative sorghum phenolic extracts at 0–200 μM GAE for up to 72 h on cell number in both HepG2 and Caco-2 cells. Cells were treated with sorghum phenolics extracted from the representative sorghum accessions PI329694 and PI570481 at various concentrations (0–200 μM GAE) for up to 72 h in 6-well plates, then detached by trypsin-EDTA solution, and the cell number was counted by hemacytometer at each timepoint. Values are expressed as a percentage of the untreated control at 24 h by Mean ± SD (*n* = 3). The significance of the trend for cell growth inhibition at various concentrations at each timepoint was analyzed with linear regression, *p* < 0.05.

As PI329694 and PI570481 had the highest phenolic content and the lowest IC_50_ value, respectively, extracts of these were selected for both HepG2 and Caco-2 treatments, resulting in a similar dose- and time-dependent reduction in cell number (Figure 2). Both IC_50_ values were much lower than that of the control F10000 (Table 2).

### 3.3. Cytotoxicity Assay

Cell viability, as assessed by lactate dehydrogenase leakage, was generally greater than 85% in the adherent cells, and the treated cells did not differ significantly from the vehicle-treated control (data not shown).

### 3.4. Cell Cycle Arrest

**Figure 3:**
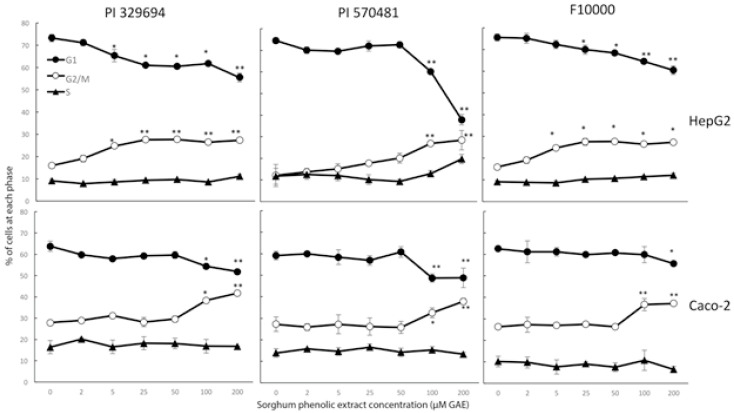
Cytostatic effect of representative sorghum phenolic extracts in HepG2 and Caco-2 cell lines. Cells were treated with sorghum phenolics extracted from the representative sorghum accessions PI329694 and PI570481 at 0–200 μM GAE for up to 72 h, and then cell cycle was monitored by a DNA flow cytometric analysis. Values are expressed as Mean ± SD (*n* = 3), * *p* < 0.05, ** *p* < 0.01 versus the vehicle control.

The treatment of either HepG2 or Caco-2 cells with the high phenolic concentrations extracted from either PI329694 or PI570481 significantly induced cell cycle arrest at G2/M phase (Figure 3). The percentage of cells at G1 phase decreased correspondingly, while the proportion of cells at S phase was not significantly altered. When compared with the control F10000, which showed a similar induction, this was actually much less potent due to there being over 20-times less phenolic content.

### 3.5. Apoptosis

**Figure 4:**
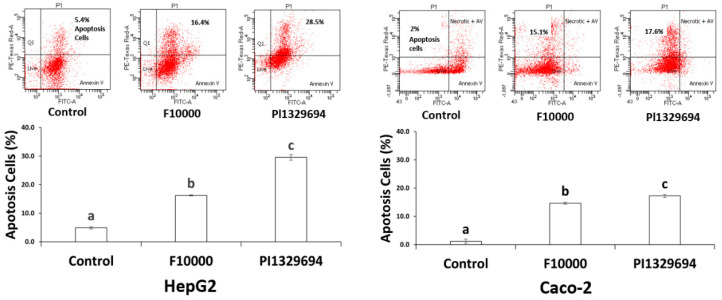
Apoptosis induced by representative sorghum phenolic extracts in HepG2 and Caco-2 cell lines. Cells were treated with sorghum phenolics extracted from the representative sorghum accessions PI329694 and F10000 at 0–200 μM GAE for up to 72 h, and then apoptosis was analyzed by FITC annexin V staining protocol. Values are expressed as Mean ± SD (*n* = 3), Means with different alphabetical letters differ significantly, *p* ≤ 0.05.

As shown in Figure 4, the treatment of either HepG2 or Caco-2 cells with the phenolic extracts at high concentrations from PI329694 or positive control F10000 resulted in a significant increase in the percentage of apoptotic cells. A significant induction by the extract of PI329694 was also observed when compared with the control F10000.

### 3.6. Correlation Coefficient

As shown in Table 3, a significant inverse correlation was observed between total phenolic contents and IC_50_ values in the extracts of all accessions (r = −0.6806, *p* < 0.05), indicating that the cell growth inhibition by the sorghum phenolic extracts was significantly associated with the phenolic content. Meanwhile, significant inverse correlations were observed between the decrease in cell number and the increase in G2/M and apoptotic cells, suggesting that the decrease in cell number by sorghum phenolic extracts was associated with cell cycle arrest and apoptosis induction.

## 4. Discussion

Thirteen specialty sorghum accessions were selected based on their high levels of phenolics when compared with the control accession F10000, which contained the fewest phenolics. Two cell lines derived from liver and colorectal cancer were used as the liver is the major site for the metabolism of dietary compounds including phenolic compounds and the intestine is the major site for the absorption of phenolic compounds. To our knowledge, this is the first study to compare the cellular impact of thirteen different sorghum phenolic extracts on both liver and intestine cancer cells, which may provide a better understanding of the anti-cancer activities of sorghum phenolics with a long-term goal of promoting sorghum products as phenolic-sustainable functional foods for health benefits.

In the present study, total phenolic contents, determined by Folin–Ciocalteu assay, were 30–64 mg GAE/g DW in the selected 13 sorghum accessions (Table 1) while the control contained only 2.3 ± 0.2 mg GAE/g DW. This result is inconsistent with our previous study [13,23].

Treatment of sorghum phenolics at various concentrations for up to 72 h resulted in a dose- and time-dependent inhibition of cell growth in both HepG2 and Caco-2 cells (Figure 1 and Figure 2). The values of IC_50_ varied from 85.8 to 221.8 mg DW/mL, with sorghum accession PI570481 being the lowest when compared with the control that showed the highest value (Table 2). Similar results were also found in Caco-2 cells when treated with various concentrations of phenolic extracts from PI329694 or PI570481 (Table 2 and Figure 2). These results indicate that, the more sorghum accessions are rich in phenolic compounds, the more they effectively suppress cancer cell growth. This result is in agreement with our previous study conducted in sorghum phenolics in HepG2 and Canc-2 cells [13,23].

To investigate the underlying mechanisms of cancer cell growth inhibition by sorghum phenolics, cytotoxicity was assessed for each concentration of phenolic extracts from various sorghum accessions in both cell lines, resulting in a difference in cytotoxicity of less than 15% between the treated and non-treated cells. It should be noted that the cytotoxicity detected did not differ significantly from the vehicle-treated control, suggesting sorghum phenolic extracts are non-toxic to the cells. In addition, the treatment of both cell lines by the phenolics extracted from PI329694 or PI570481 showed an induction of cell cycle arrest at G2/M phase (Figure 3) and apoptotic cells (Figure 4). These results indicate that the inhibition of cell growth by sorghum phenolic compounds appears to be cytostatic but not cytotoxic. The cell cycle arrest induced by sorghum phenolics may trigger the DNA repair machine, leading to apoptosis, as confirmed by our apoptosis analysis, suggesting that treatment of both cell lines with sorghum phenolic extracts significantly induced the apoptotic cells compared with the vehicle control.

Coefficient correlations were further calculated between total phenolic content and IC_50_ values, cell number and cell cycle arrest at G2/M phase, and cell number and proportion of apoptotic cells. Significant inverse correlations observed between total phenolic contents and IC_50_ values in thirteen sorghum accessions (Table 3) suggest that the cell growth inhibition by sorghum extracts is directly associated with their phenolic content. As strong significant inverse correlations were also observed between cell number and the accumulation of cell cycle arrest at G2/M phase and apoptosis in both cell lines (Table 3), the inhibitory effect of sorghum phenolic compounds on cell growth appears to be through a cytostatic mechanism.

## 5. Conclusions

In conclusion, the present study showed that phenolic extracts in various sorghum accessions effectively inhibited HepG2 or Caco-2 cancer cell growth in a dose- and time- dependent manner. The cell growth inhibition by the sorghum phenolic extracts was significantly associated with their phenolic content. Furthermore, the inhibition appeared to be mediated by cytostatic and apoptotic mechanisms rather than cytotoxicity. Taken together, this study is the first to investigate and compare the anti-cancer effect of sorghum phenolic compounds in both HepG2 and Caco-2 cell lines. The results suggest that sorghum is a valuable food and crop with health benefits to provide sustainable phenolics for potential cancer prevention.

## Figures and Tables

**Table 1:** Phenolic Contents in Various Specialty Sorghum Accessions (mean ± SD, *n* = 3).

Sorghum Accession No.	Phenolic Contents (mg GAE/g DW)
F10000 (control)	2.3 ± 0.2
PI 559855	31.0 ± 0.2
PI 152687	44.6 ± 2.9
PI 570819	48.2 ± 4.4
PI 570993	51.0 ± 1.9
PI 559733	51.5 ± 1.4
PI 570366	54.3 ± 1.7
PI 152653	54.6 ± 1.2
PI 570484	54.8 ± 0.8
PI 193073	55.1 ± 4.5
PI 570889	58.0 ± 2.0
PI 568282	58.3 ± 2.5
PI 570481	58.5 ± 2.5
PI 329694	63.7 ± 2.5

**Table 2:** IC_50_ Values of specialty sorghum phenolic extracts in HepG2 and Caco-2 cells.

Sorghum Accession No.	IC_50_^a^ (mg DW/mL)	
	HepG2	Caco-2
F10000 (control)	1275.6	1131.3
PI 559855	221.8	
PI 152687	138.9	
PI 570819	192.1	
PI 570993	146.2	
PI 559733	90.8	
PI 570366	120.9	
PI 152653	127.9	
PI 570484	158.0	
PI 193073	177.3	
PI 570889	120.9	
PI 568282	113.3	
PI 570481	85.8	115.6
PI 329694	126.8	102.4

^a^ Means ± SD, *n* = 3.

**Table 3:** Correlation coefficient (r) between phenolic contents and IC_50_ values from the phenolic extracts of 13 sorghum accessions in HepG2 cells, or between cell number and cell cycle arrest or apoptosis from control, PI329694 and PI570481 in both cell lines.

	IC_50_		
**Total phenolics**	−0.6806 *		
		**Cell Arrest at G2/M**	**Apoptosis Cells**
**HepG2**			
F10000 control		−0.8608 *	−0.9377 **
PI329694	**Cell number**	−0.8281 *	−0.9764 **
PI570481		−0.9469 **	
**Caco-2**			
F10000 control		−0.7599 *	−0.9719 **
PI329694	**Cell number**	−0.7655 *	−0.8199 *
PI570481		−0.6840 *	

* *p* < 0.05, ** *p* < 0.01.

## Data Availability

The data is contained within the article.
